# Phylogenomic evidence for ancient recombination between plastid genomes of the *Cupressus*-*Juniperus*-*Xanthocyparis* complex (Cupressaceae)

**DOI:** 10.1186/s12862-018-1258-2

**Published:** 2018-09-10

**Authors:** Andan Zhu, Weishu Fan, Robert P. Adams, Jeffrey P. Mower

**Affiliations:** 1 Center for Plant Science Innovation, University of Nebraska, Lincoln, NE 68588 USA; 2 Department of Agronomy and Horticulture, University of Nebraska, Lincoln, NE 68583 USA; 3 Germplasm Bank of Wild Species, Kunming Institute of Botany, Chinese Academy of Sciences, Kunming, 650201 Yunnan China; 4 Biology Department, Baylor University, Waco, TX 76798 USA

**Keywords:** *Callitropsis*, *Cupressus*, *Juniperus*, *Hesperocyparis*, *Xanthocyparis*, Cupressaceae, Plastid genome, Phylogenetic incongruence, Introgression, Recombination

## Abstract

**Background:**

Phylogenetic relationships among Eastern Hemisphere cypresses, Western Hemisphere cypresses, junipers, and their closest relatives are controversial, and generic delimitations have been in flux for the past decade. To address relationships and attempt to produce a more robust classification, we sequenced 11 new plastid genomes (plastomes) from the five variously described genera in this complex (*Callitropsis*, *Cupressus*, *Hesperocyparis*, *Juniperus*, and *Xanthocyparis*) and compared them with additional plastomes from diverse members of Cupressaceae.

**Results:**

Phylogenetic analysis of protein-coding genes recovered a topology in which *Juniperus* is sister to *Cupressus*, whereas a tree based on whole plastomes indicated that the *Callitropsis*-*Hesperocyparis*-*Xanthocyparis* (CaHX) clade is sister to *Cupressus*. A sliding window analysis of site-specific phylogenetic support identified a ~ 15 kb region, spanning the genes *ycf1* and *ycf2*, which harbored an anomalous signal relative to the rest of the genome. After excluding these genes, trees based on the remainder of the genes and genome consistently recovered a topology grouping the CaHX clade and *Cupressus* with strong bootstrap support. In contrast, trees based on the *ycf1* and *ycf2* region strongly supported a sister relationship between *Cupressus* and *Juniperus*.

**Conclusions:**

These results demonstrate that standard phylogenomic analyses can result in strongly supported but conflicting trees. We suggest that the conflicting plastomic signals result from an ancient introgression event involving *ycf1* and *ycf2* that occurred in an ancestor of this species complex. The introgression event was facilitated by plastomic recombination in an ancestral heteroplasmic individual carrying distinct plastid haplotypes, offering further evidence that recombination occurs between plastomes. Finally, we provide strong support for previous proposals to recognize five genera in this species complex: *Callitropsis*, *Cupressus*, *Hesperocyparis*, *Juniperus*, and *Xanthocyparis*.

**Electronic supplementary material:**

The online version of this article (10.1186/s12862-018-1258-2) contains supplementary material, which is available to authorized users.

## Background

The discovery in northern Vietnam of a new conifer species, *Xanthocyparis vietnamensis* Farjon & T. H. Nguyên [1, 2], has caused taxonomic upheaval within the Cupressaceae. Based on distinctive morphological traits, this conifer was initially placed in a new genus (*Xanthocyparis*) and inferred to be closely related to *Callitropsis nootkatensis* (D. Don) Oersted ex D. P. Little [1]. *Ca. nootkatensis* is another taxonomically controversial species that has been variously classified into *Chamaecyparis*, *Callitropsis*, *Cupressus*, and *Xanthocyparis* [3, 4]. How these two species relate to one another and to other Cupressaceae conifers has been a topic of ongoing taxonomic debate, driven by a paucity of distinguishing morphological characteristics [1, 3, 5] as well as incongruence among molecular phylogenetic analyses [4, 6–14]. From a broader perspective, the phylogenetic positions of *X. vietnamensis* and *Ca.* *nootkatensis* impinge on a large taxonomic debate regarding the treatment of Western Hemisphere cypresses (hereafter *Hesperocyparis*) and Eastern Hemisphere cypresses (hereafter *Cupressus*) [8, 9, 15], and affect phylogeographic interpretations of migration patterns among flora spanning the Eastern and Western Hemispheres [9, 10].

Phylogenetic relationships among the (up to) five recognized genera (*Callitropsis*, *Cupressus*, *Hesperocyparis*, *Juniperus*, *Xanthocyparis*) of this (hereafter CaCuHJX) complex of Cupressaceae species are unresolved. Early phylogenetic studies based primarily on the internal transcribed spacer region of the nuclear ribosomal DNA cluster have generally recovered a tree in which *X. vietnamensis* and *Ca.* *nootkatensis* form a clade that is sister to *Hesperocyparis*, which together are more closely related to *Juniperus* than to *Cupressus* [4, 6–8, 13]. In contrast, chloroplast markers, while generally providing less resolution, have tended to construct (or at least be consistent with) a grouping of *Ca.* *nootkatensis* and *Hesperocyparis*, which are successively sister to *X. vietnamensis*, then *Cupressus*, and finally *Juniperus* [4, 7–11, 13, 14]. Analyses using nuclear or mitochondrial protein-coding genes [7, 12], or the fastest-evolving sites in the plastid genome [14], have recovered a third topology, in which *Juniperus* is monophyletic with *Cupressus* while *Ca.* *nootkatensis, X. vietnamensis*, and *Hesperocyparis* form a second monophyletic group with less certain resolution.

Collectively, all of the aforementioned studies agree that *Hesperocyparis* is more closely related to *Ca.* *nootkatensis* and *X. vietnamensis* than to *Cupressus* or *Juniperus*, although the precise relationships among these five genera are as yet unclear. Intriguingly, these previous studies also suggest fundamental incongruence between and within the plastid and nuclear genomes. To stabilize the classification of these five genera, and to explore the source of conflicting intraplastomic signals, we sequenced 11 plastomes and compared them with 10 existing plastomes from all five genera. Through extensive phylogenetic comparisons, we present a robust phylogeny of the five genera and identify the genes *ycf1* and *ycf2* as the major source of intraplastomic phylogenetic conflict. By integrating recent discoveries on organelle inheritance, we highlight potential effects of genetic leakage and ancient recombination on phylogenomic analysis.

## Results

### General features of newly sequenced Cupressaceae plastomes

**Fig. 1:**
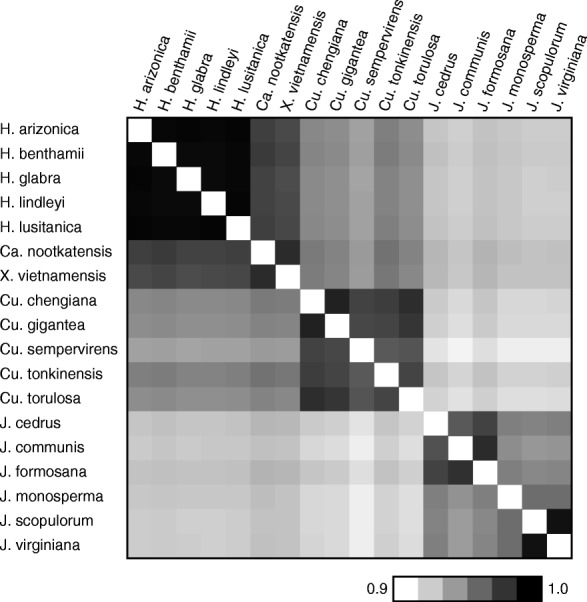
Similarity plot based on pairwise comparison of plastomes from the untrimmed whole-genome alignment. Similarity scores are color coded from white (90% sequence identity) to black (100% sequence identity)

We sequenced complete plastomes from 11 species spanning five genera of Cupressaceae, including *Callitropsis* (*Ca.* *nootkatensis*), *Cupressus* (*Cu. sempervirens*, *Cu. tonkinensis*, *Cu. torulosa*), *Hesperocyparis* (*H. arizonica*, *H. benthamii*, *H. glabra, H. lindleyi*, *H. lusitanica*), *Juniperus* (*J. communis*) and *Xanthocyparis* (*X. vietnamensis*). Genomes are very similar in size (127–129 kb) and content, with nearly identical proportions of guanine plus cytosine (G + C = 34.6–34.9%) and an identical set of 82 protein-coding genes, 4 ribosomal RNAs, 33 transfer RNAs and 18 introns (Table 1). Pairwise alignment of entire plastome sequences demonstrated a high level of intra- and intergeneric similarity (Fig. 1). Notably, the plastomes from *Cupressus* and the CaHX clade are in all cases more similar to one another (93.6–95.5% identity) than they are to *Juniperus* plastomes (90.5–93.0%).

**Table 1:** General features of newly sequenced Cupressaceae plastomes

Species	Genome size (bp)	Protein genes	Introns	G + C (%)	IR size (bp)	Depth of coverage
*Ca.* *nootkatensis*	127,150	82	18	34.7	266	441
*Cu. sempervirens*	129,150	82	18	34.6	264	358
*Cu. tonkinensis*	127,835	82	18	34.7	255	142
*Cu. torulosa*	128,322	82	18	34.6	271	221
*H. arizonica*	127,158	82	18	34.7	268	451
*H. benthamii*	127,007	82	18	34.7	269	176
*H. glabra*	126,993	82	18	34.7	268	532
*H. lindleyi*	127,004	82	18	34.7	271	213
*H. lusitanica*	127,113	82	18	34.7	270	190
*J. communis*	127,904	82	18	34.9	272	281
*X. vietnamensis*	127,541	82	18	34.7	266	325

### Different plastid phylogenomic approaches construct strongly conflicting trees

**Fig. 2:**
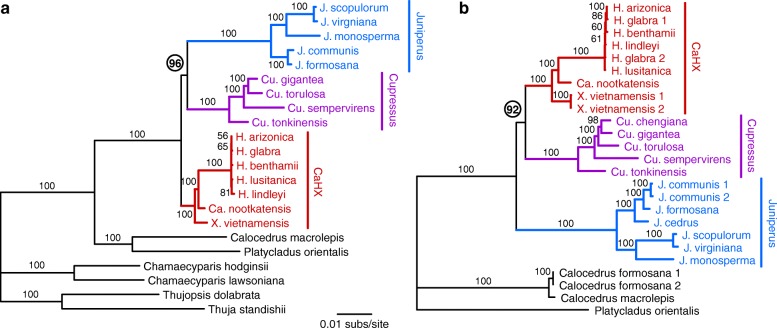
Phylogenomic analyses. **a** Results from the 82-gene alignment. **b** Results from the whole genome alignment. Circled bootstrap values indicate the major point of incongruence between the two trees

To examine the phylogenetic relationships among *Callitropsis*, *Cupressus*, *Hesperocyparis*, *Juniperus*, and *Xanthocyparis*, we performed plastid phylogenomic analyses using two common approaches: 1) a concatenated alignment of all 82 protein-coding genes, and 2) a whole plastome alignment. The trees resulting from analysis of both data sets were largely congruent, particularly with respect to the relationships among species within *Juniperus*, within *Cupressus*, and among genera within the CaHX clade (Fig. 2).

However, there was strong conflict for relationships among the *Cupressus, Juniperus* and CaHX clades (Fig. 2). The tree constructed from the 82-gene alignment indicated a sister relationship between *Juniperus* and *Cupressus* with strong (96%) bootstrap support (Fig. 2a). In contrast, the whole plastome alignment resulted in a tree that united the CaHX and *Cupressus* clades with strong (92%) bootstrap support (Fig. 2b). For each data set, the Approximately Unbiased and Shimodaira-Hasegawa alternative topology tests significantly rejected (*p* < 0.05) the topology recovered by the other data set. Thus, two standard phylogenomic approaches produced strongly supported but incongruent trees.

### *ycf1* and *ycf2* have a distinct phylogenetic signal relative to the rest of the plastome

**Fig. 3:**
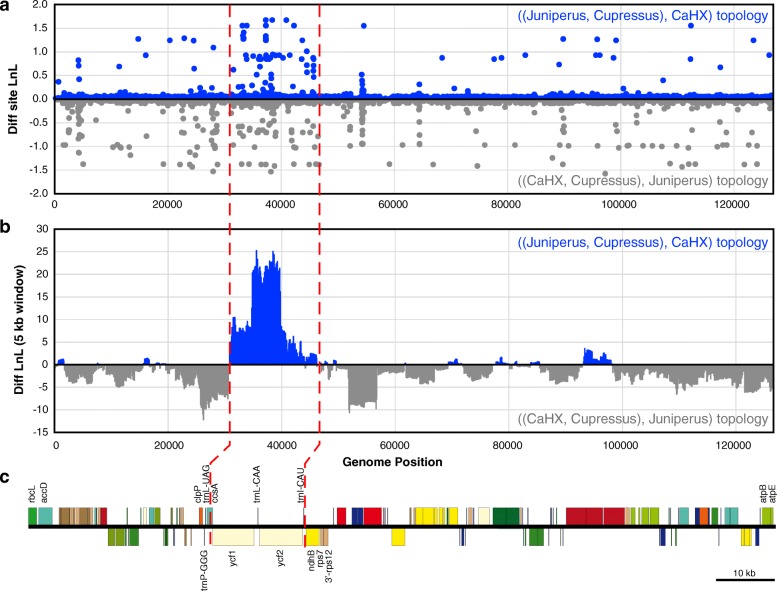
Distinct phylogenetic signals in Cupressaceae plastomes. **a** Difference in site log likelihoods for the two major tree topologies recovered in Fig. 1. Sites supporting the *Juniperus* + *Cupressus* topology are shown in blue, while sites supporting the CaHX + *Cupressus* topology are shown in gray. **b** Sliding window analysis (window size = 5000; step size = 100) showing the sum of the difference in site log likelihoods in segments of the genome. **c** Linear map of the *X. vietnamensis* plastome. Genes placed above or below the map indicate that they are on opposite strands of the genome sequence. Red dotted lines demark the segment of the genome that exhibits an anomalous phylogenetic signal relative to the rest of the genome

To investigate the source of phylogenetic incongruence within the plastome, we calculated the likelihood of each site in the whole plastome alignment for the two competing topologies. By taking the difference in the log of the site-likelihood values for the two tree topologies, we identified those sites that provided the strongest preference for one or the other topology. Sites providing strong preference for the *Juniperus* + *Cupressus* topology were mostly clustered within the 31 kb to 47 kb segment in the whole plastome alignment, whereas sites providing strong support for the CaHX + *Cupressus* topology were more evenly spread throughout the data set (Fig. 3a). Sliding window analysis provided clear evidence that this 31 kb to 47 kb genomic segment favored the *Juniperus* + *Cupressus* relationship, whereas the remainder of the genome provided greater support for the CaHX + *Cupressus* relationship (Fig. 3b). This anomalous region of the alignment corresponds to a segment of the plastome containing the entirety of the genes *ycf1*, *trnL-CAA*, *ycf2*, and *trnI-CAU* and a portion of the *ccsA* gene (Fig. 3c).

**Fig. 4:**
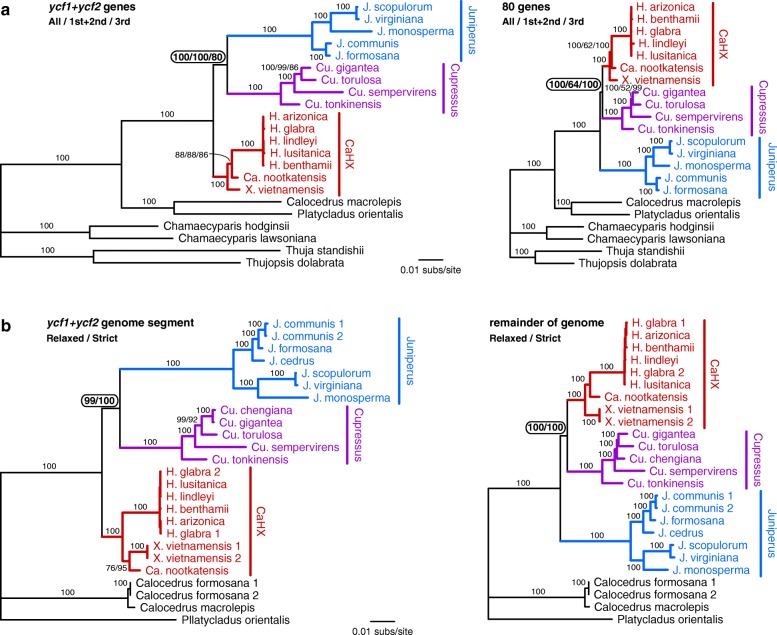
Phylogenetic analyses using separated data sets. **a** Results from the *ycf1* and *ycf2* genes (left) and the remaining 80 genes (right). **b** Results from the section of the whole genome alignment containing the *ycf1* and *ycf2* genes (left) and the remaining portion of the whole genome alignment (right). Circled bootstrap values indicate the major point of incongruence among the trees

To verify that *ycf1* and *ycf2* have an anomalous phylogenetic signal, we reevaluated the concatenated gene alignment after separating the *ycf1* + *ycf2* genes from the remaining 80 genes (Fig. 4a). We also reexamined the whole-plastome analyses with the *ycf1* + *ycf2* genomic region separated from the remainder of the genome (Fig. 4b). Results of both analyses were fully consistent. The *ycf1* + *ycf2* gene and genomic segment data sets provided strong support for *Cupressus* + *Juniperus* as sister taxa, while the rest of the genes and genome produced trees with strong support for CaHX + *Cupressus* (Fig. 4a and b).

The *ycf1* and *ycf2* genes are known to be fast evolving, with substantial levels of positive selection and numerous indels [16–18]. In Cupressaceae, *ycf1* and *ycf2* are also relatively faster evolving, as demonstrated by the generally 2- to 3-fold longer branch lengths in the trees of *ycf1 + ycf2* relative to the remaining genes (Fig. 4a) and genomic regions (Fig. 4b), and by the larger number of gap-containing columns in the untrimmed *ycf1* + *ycf2* gene alignment (22.2% of 16,614 positions) compared with the untrimmed 80-gene alignment (5.50% of 62,853 positions). Despite the faster relative rate of evolution, no substitutional saturation was detected (See Additional file 1: Table S1) in the Gblocks-trimmed *ycf1 + ycf2* data sets based on an entropy test of substitution saturation [19, 20].

We also confirmed that the different selection pressures and rates of evolution at 3rd codon positions compared with 1st and 2nd codon positions had no effect on the recovered topology. Indeed, regardless of codon partitioning scheme (all codon positions, 1st + 2nd positions only, or 3rd positions only), the *ycf1* + *ycf2* gene data sets recovered *Cupressus* + *Juniperus* with moderate to strong support, while the 80 gene data set recovered *Cupressus* + CaHX with moderate to strong support (Fig. 4a). Finally, given the large number of indels in the *ycf1* and *ycf2* alignments, we examined the effect of gap treatment during alignment filtering of the genome data sets. Regardless of Gblocks settings, the *ycf1* + *ycf2* genomic segment recovered *Cupressus* + *Juniperus* with strong support, while the remainder of the genome recovered *Cupressus* + CaHX with strong support (Fig. 4b).

### Structural features of Cupressaceae plastomes

**Fig. 5:**
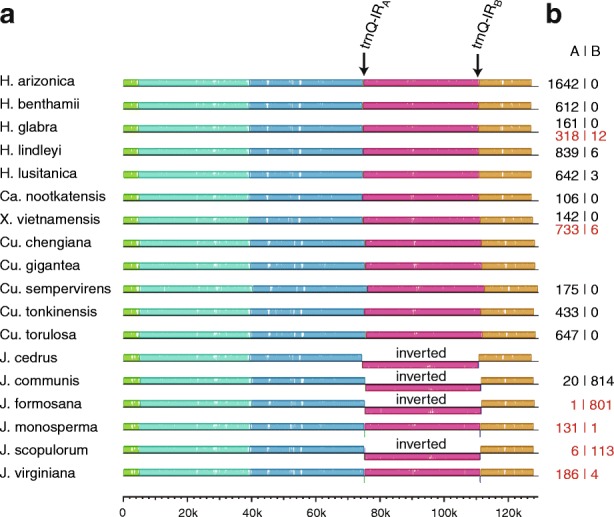
Analysis of structural variation among Cupressaceae plastomes. **a** progressiveMAUVE plot of whole plastome sequences. The location of the two *trnQ*-IR copies is marked by arrows, and species containing the intervening sequence in an inverted orientation are labeled. **b** Numbers of read pairs spanning the *trnQ*-IR that provide support for the genome in either the “A” or “B” arrangement. Numbers listed in red are from previous studies [21, 24]

Cupressaceae plastomes lack the large inverted repeat (IR) that is a diagnostic feature of most other land plant plastomes. Instead, they contain a much smaller (~ 260 bp) IR that duplicates the *trnQ* gene [21–23]. The two copies of the *trnQ*-IR flank a ~ 36 kb segment of the plastome, and collinearity analysis indicated that IR recombination has led to the inversion of this genomic segment in the newly sequenced *J. communis* plastome (Fig. 5a). This inverted region was previously defined as the “B” arrangement to contrast with the non-inverted “A” arrangement that is present in most Cupressaceae species, although several other Cupressaceae species were also shown to have a plastome in this “B” arrangement [21].

Analysis of mapped read pairs (Fig. 5b) verified that nearly all read pairs that span the *trnQ-*IR (814/834) supported the “B” arrangement in *J. communis*. However, 2.7% (20/834) of these *J. communis* read pairs instead supported the existence of the “A” arrangement, demonstrating that the “A” arrangement exists at a substoichiometric level relative to the predominant “B” arrangement within the sampled *J. communis* individual. By contrast, the *H. lindleyi* and *H. lusitanica* plastomes exist primarily in the “A” arrangement in the sampled individuals, with a small proportion (< 1%) of reads supporting the presence of the “B” arrangement at a substoichiometric level. The coexistence of predominant and substoichiometric forms of the plastome was previously reported [21, 24] for other Cupressaceae species (Fig. 5b, shown in red).

## Discussion

Previous studies have disagreed on the inferred phylogenetic relationships among major lineages of the CaCuHJX clade, which comprises Eastern Hemisphere cypresses (*Cupressus*), Western Hemisphere cypresses (*Hesperocyparis*), junipers (*Juniperus*), and the taxonomically enigmatic species *X. vietnamensis* and *Ca.* *nootkatensis*. Their relationships have remained contentious due in part to phylogenetic incongruence between nuclear and plastid data as well as intragenomic conflict among loci within the plastid and nuclear genomes. In this study, 21 complete plastomes (11 newly generated) from species in the CaCuHJX complex were used to reexamine phylogenetic relationships among genera and to evaluate the distribution of conflicting phylogenetic signals across the plastome. Our whole-plastome analyses offer substantially more informative characters than previous analyses using a small number of loci [4, 6–13] and more than twice the number of ingroup taxa compared with the only other plastome-based phylogenetic study [14].

Our results demonstrate that different phylogenomic approaches can produce strongly supported but conflicting phylogenetic hypotheses (Fig. 2). In this case, we showed that the phylogenetic conflict comes from a ~ 15 kb region of the plastome (spanning *ycf1* and *ycf2*) that exhibits a phylogenetic signal incongruent with the rest of the plastome (Figs. 3 and 4). Phylogenetic incongruence of one or few loci within the plastid genome has been reported in other lineages of seed plants, including Sileneae [25], *Citrus* [26], *Pinus* [16] and *Picea* [18], with the incongruence also spanning a region containing the *ycf1* and *ycf2* genes for the latter two genera in Pinaceae. An important question arising from these analyses is why some plastid loci may have distinct evolutionary signals. Below we discuss the potential causes and taxonomic significance of these findings.

### Unique characteristics of *ycf1* and *ycf2* do not explain phylogenetic incongruence

There is no doubt that *ycf1* and *ycf2* exhibit higher rates of sequence and indel evolution compared with most plastid genes. Both the *Pinus* and *Picea* studies [16, 18] identified several sites of the *ycf1* and *ycf2* genes under positive selection. However, pervasive positive selection is not likely to be a determining factor for the conflicting phylogenetic trees in the CaCuHJX complex. The codon partitioning results argues strongly against any confounding phylogenetic effects stemming from differences in substitution rate or selection pressure at different codon positions (Fig. 4a). Second, while the *ycf1* and *ycf2* genes are mutational hotspots for the accumulation of indels, analysis of data sets that either excluded all gaps (strict filtering) or allowed gaps when present in < 50% of taxa (relaxed filtering) recovered the same tree, which was still incongruent with signals of the rest of the genome (Fig. 4b).

Finally, the effect of substitutional saturation can be ruled out because individual branch lengths in all trees are very short at this low taxonomic level (Fig. 4) and no substitutional saturation was detected by an entropy test [19, 20] implemented in DAMBE. Note that the previous plastome-based study of the CaCuHJX complex did report substitutional saturation for nine plastid genes (including *ycf1* and *ycf2*) [14]; in that study, untrimmed alignments were apparently used for the entropy analysis based on the fact that we can somewhat replicate their results when using our own untrimmed alignment of the *ycf1* + *ycf2* gene data set (See Additional file 1: Table S1). However, given the high indel rate in the *ycf1* and *ycf2* genes, alignment filtration using programs such as Gblocks is a necessity to avoid spurious results in phylogenetic analysis, and this would also apply to entropy tests which aim to assess the suitability of a data set for phylogenetic analysis. Moreover, the DAMBE software warns against including gaps and unresolved characters in the alignment due to the potential for false positives.

### A biological basis for phylogenetic incongruence

If phylogenetic artifacts due to the unique properties of the *ycf1* and *ycf2* genes can be excluded, then biological factors may be the more likely source of phylogenetic incongruence. To explain the intragenomic conflict within the plastomes of the CaCuHJX clade, we propose that the anomalous signal resulted from an ancient introgression event involving the *ycf1* and *ycf2* genes. This event would require several evolutionary processes to occur: 1) ancient hybridization or incomplete lineage sorting to establish an ancestral population having two plastid haplotypes with distinct evolutionary ancestry, 2) creation of a heteroplasmic individual containing both plastid haplotypes via at least occasional biparental inheritance, and 3) recombination between the two plastid haplotypes.

Hybridization is a common phenomenon in plant evolution that can confound phylogenetic analyses, particularly when using cytoplasmic loci [27], and even more so if recombination among distinct plastid haplotypes has occurred [28]. In conifers, hybridization has resulted in chloroplast capture, nuclear introgression, and phylogenetic incongruence between the nuclear and plastid genomes [18, 29, 30]. Thus, it is plausible that members of the CaCuHJX complex may have experienced some level of reticulate evolution. In fact, long-distance dispersal of seed cones has been well documented for many *Juniperus* species [9, 10], and ancient hybridization has been previously suggested to explain phylogenetic incongruence between the nuclear and plastid genomes in the CaCuHJX clade [12]. Incomplete lineage sorting could also be an explanation for coexisting plastome haplotypes in a population, although this mechanism has received less attention in the plastome literature [31, 32].

Once distinct plastome haplotypes were established in a population (via ancient hybridization or incomplete lineage sorting), some level of biparental inheritance could have created a heteroplasmic state, which could then have facilitated recombination between plastomes from different species, resulting in the introgression of foreign *ycf1* and *ycf2* genes. Frequent reversals of uniparental inheritance (maternal-to-paternal and vice versa) have been found for both mitochondrial and chloroplast genomes [33], and genetic leakage has been observed in many Cupressaceae species (See Additional file 1: Table S2) and other seed plants [34–36]. Heteroplasmy and recombination could neatly explain the anomalous phylogenetic signal that is confined to the ~ 15 kb region of the plastome, regardless of the fast-evolving properties of the two *ycf* genes.

The anomalous grouping of *Juniperus* and *Cupressus* in the *ycf1* + *ycf2* analyses suggests that the ancient introgression of the *ycf1* and *ycf2* genomic segment occurred between these two lineages. The crown group ages for *Cupressus* and *Juniperus* have been dated to ~ 30 and ~ 40 million years, respectively, while the crown group age for the entire CaCuHJX clade was estimated to be ~ 60 million years [9]. These dates suggest that the ancient hybridization and recombination event probably occurred 40–60 million years ago, subsequent to the initial diversification of the CaCuHJX clade but prior to the diversification of the *Cupressus* and *Juniperus* lineages. However, the direction of *ycf1* + *ycf2* introgression (from *Cupressus* to *Juniperus* or from *Juniperus* to *Cupressus*) cannot be determined from the available data.

### Taxonomic implications of phylogenetic results

Except for the intragenomic conflict observed in our plastomic data regarding the relationships among the *Cupressus*, *Juniperus*, and CaHX clades, phylogenetic results are otherwise largely congruent in the trees based on protein-coding genes and complete plastomes. Importantly, all data sets but one from this study strongly support a sister group relationship between *Callitropsis* and *Hesperocyparis* within the CaHX clade (Fig. 2; Fig. 4), which is generally consistent with previous studies using at least 10 kb of sequence data [9–11, 13, 14]. The lone contrasting data set (*ycf1* + *ycf2* genomic data) instead supports a sister group relationship between *Ca.* *nootkatensis* and *X. vietnamensis* (Fig. 4b, left), which has also been observed in a minority of previous studies, primarily based on nuclear internal transcribed spacer data [4, 7, 8]. Nevertheless, the weight of evidence from this study and others indicates that *Ca.* *nootkatensis* and *X. vietnamensis* are not sister taxa; thus, the previous suggestion [3] to classify both species into separate monotypic genera appears well justified.

Finally, alternative suggestions to treat the entire CaHX clade as a single genus *Callitropsis* [7], or to maintain a more broadly defined *Cupressus* *sensu* *lato* (*s.l.*) that includes the CaHX clade [15], are problematic. The maintenance of *Cupressus s.l.* is problematic due to uncertainty in the placement of *Juniperus*. Notably, a paraphyletic *Cupressus s.l.* is consistently recovered in the few studies that have utilized nuclear or mitochondrial protein-coding genes [7, 8, 12, 13] as well as a minority of plastid analyses from this (Fig. 2; Fig. 4) and other [14] studies; more nuclear and mitochondrial data is required to explore this issue further. Furthermore, while the CaHX clade is clearly monophyletic in this and many previous studies, there are a variety of morphological characters that distinguish *Hesperocyparis* from *Ca.* *nootkatensis* and *X. vietnamensis* [8], arguing against circumscribing all three genera into a single, more broadly defined genus. Collectively, while there is still room for debate on the precise relationships among species in the CaCuHJX clade of Cupressaceae, the weight of evidence strongly favors recognition of five separate genera: *Callitropsis*, *Cupressus*, *Hesperocyparis*, *Juniperus*, and *Xanthocyparis*.

## Conclusions

Our results provide further evidence that standard phylogenomic analyses can produce strongly supported but conflicting trees, implying that phylogenomic results should be performed in multiple ways with different data partitioning schemes to unmask potential signals of conflict. In our study, we showed that the conflicting phylogenetic signal was localized to the *ycf1* and *ycf2* region of the genome, which we suggest was due to introgression of this region in an ancestor of this species complex. This hypothesis implies that plastomic recombination must have occurred between distinct haplotypes that coexisted in an ancestral heteroplasmic individual. Finally, after exclusion of the introgressed *ycf1* and *ycf2* genes from the data sets, our analyses recovered a robust phylogeny of the five genera and provided strong evidence in support of previous proposals to recognize five distinct genera in this species complex: *Callitropsis*, *Cupressus*, *Hesperocyparis*, *Juniperus*, and *Xanthocyparis*.

## Methods

### Sample collection and DNA sequencing

Leaf samples (50 mg each) from mature trees (*Ca*. *nootkatensis*, *Cu. sempervirens*, *H. arizonica*, *H. benthamii*, *H. glabra*, *H. lindleyi*, *H. lusitanica*, and *J. communis*) were collected on roadsides in common areas of public land. Leaf samples (50 mg each) from remaining samples (*Cu. tonkinensis, Cu. torulosa*, and *X. vietnamensis*) were collected from seedlings grown by Keith Rushforth (UK) in his garden from seeds collected by him. Thus, no samples were subject to institutional, national or international guidelines for collection. DNAs were extracted according to procedures described previously [21] and sequenced on the Illumina HiSeq 2500 platform at BGI (Shenzhen, China) or the Illumina MiSeq system at the Center for Genomics and Bioinformatics at Indiana University (Bloomington, IN). Details of collection sites, voucher numbers, and sequencing results are provided (See Additional file 1: Table S3).

### Plastome assembly and annotation

Plastomes were assembled using an established procedure [21, 37, 38]. For each species, a draft sequence was assembled from raw reads using Velvet version 1.2.03 [39] with pairwise combinations of different Kmer values (61, 71, 81, 91, 101) and expected coverage values (50, 100, 200, 500, 1000), and a final consensus sequence was generated from at least three independent assemblies. Genes were initially annotated using DOGMA [40], followed by manual correction of start and stop codons based on comparison to homologs from other Cupressaceae plastomes.

### Gene and whole genome alignments

A total of 82 plastid protein-coding genes were extracted from the 11 genomes newly sequenced in this study plus additional species of Cupressaceae (See Additional file 1: Table S4). For each gene, a codon-based alignment was generated by aligning amino acid sequences with MUSCLE [41] and reverse translating the alignments into nucleotide sequences using PAL2NAL [42]. A concatenated plastid data matrix was built with FASconCAT version 1.0 [43]. The aligned 82-gene data set was 79,479 bp in length.

Whole plastome sequence alignments were also constructed from the 11 genomes newly sequenced in this study plus additional species of Cupressaceae (See Additional file 1: Table S4). First, a collinearity plot was generated with the progressiveMAUVE algorithm [44] using full genome sequences. When necessary, genomes were adjusted to start on the *rbcL* gene to ensure a consistent starting point for this plot. Next, whole genome alignments were performed with MAFFT version 7.245 [45] using the fftnsi setting. To facilitate this whole plastome alignment, the orientation of an inverted segment in some Cupressaceae plastomes (mediated by a small *trnQ*-containing inverted repeat element termed *trnQ*-IR [21]) was manually reverted such that all examined genomes were globally collinear. Plastomes from more distant outgroups were more highly rearranged and were thus excluded from the whole plastome alignments. The aligned plastome data set was 144,492 bp in length.

The aligned gene and genome data sets were trimmed using Gblocks version 0.91b [46] with default strict settings J(b1 = 13, b2 = 21, b3 = 8, b4 = 10, b5 = none) or with more relaxed settings (b1 = 13, b2 = 13, b3 = 8, b4 = 5, b5 = half). The final 82-gene data set was trimmed in codon mode (t = c) to 74,772 bp (relaxed) or 71,871 bp (strict), while the whole plastome data set was trimmed in DNA mode (t = d) to 126,645 bp (relaxed) or 113,387 bp (strict).

### Phylogenetic analysis and alternative topology tests

Phylogenetic analyses were performed using the maximum likelihood approach in PhyML version 3.0 [47] under the GTR + G + I model with 100 bootstrap replicates. The shape of the gamma distribution of rate variation, proportion of invariant sites, and substitution rate parameters were estimated during the analysis. Two competing phylogenetic hypotheses of the relationships among *Callitropsis*, *Cupressus*, *Hesperocyparis*, *Juniperus* and *Xanthocyparis* were examined using the Shimodaira-Hasegawa test and the Approximately Unbiased test, as implemented in CONSEL [48]. One topology forced *Cupressus* to be sister to *Juniperus*, while the second topology forced *Cupressus* as sister to the CaHX clade.

### Assessment of phylogenetic incongruence in the plastome

To assess levels of substitutional saturation in the data sets, saturation tests were performed on untrimmed and trimmed data sets using an entropy test based on an index of substitution saturation [19, 20] as implemented in DAMBE version 6.4.110 [49]. To examine phylogenetic signals among genomic regions, log-likelihoods for each site in the whole genome alignment were calculated on the two major topologies: *Cupressus* sister to *Juniperus* versus *Cupressus* sister to CaHX. Site likelihoods for each topology were reported in PhyML, and then the difference in log-likelihoods at each site was plotted along the genome. A sliding window analysis was performed (window size = 5000, step size = 100) that summed the difference in site likelihoods in order to show localized variation in likelihoods across 5 kb segments of the alignment.

## Additional file

Additional file 1: Supplementary Tables S1–S4. (PDF 97 kb)

## Abbreviations

CaCuHJX: *Callitropsis* + *Cupressus* + *Hesperocyparis* + *Juniperus* + *Xanthocyparis* clade
CaHX: *Callitropsis* + *Hesperocyparis* + *Xanthocyparis* clade
G + C: Guanine plus cytosine
IR: Inverted repeat
*s.l*: *sensu* *lato*
