# Supplementary material for: Phylogenomic evidence for ancient recombination between plastid genomes of the Cupressus-Juniperus-Xanthocyparis complex (Cupressaceae)
Source: BMC Evol Biol. 2018 Sep 10;18:137. doi: 10.1186/s12862-018-1258-2 (PMC6131872; doi:10.1186/s12862-018-1258-2)
Supplement: Supplementary file 1 — Supplementary Tables S1–S4. (PDF 97 kb) [file 12862_2018_1258_MOESM1_ESM.pdf]

Table S1. Evaluation of substitution saturation based on the DAMBE entropy test

		Index of substitution saturation (I_{ss}) [b]			
**Alignment**	**Alignment filter** [a]	**Alignment**	**Critical (sym)**	**Critical (asym)**	**Interpretation**
ycf1-ycf2	DAMBE	0.134	0.854	0.641	not saturated
ycf1-ycf2	Gblocks relaxed	0.211	0.856	0.641	not saturated
ycf1-ycf2	None	0.659	0.857	0.642	saturated only if tree is very unbalanced
80 gene	DAMBE	0.034	0.824	0.668	not saturated
80 gene	Gblocks relaxed	0.042	0.824	0.668	not saturated
80 gene	None	0.163	0.824	0.668	not saturated

[a] DAMBE filters all columns with gaps or ambiguities; Gblocks (relaxed) filters columns with gaps in >50% of taxa

[b] DAMBE reports critical values for fully balanced (symmetrical) and fully unbalanced (asymmetrical) trees

Table S2. Inheritance of chloroplasts and mitochondria in conifers

Species	Mt	Cp	Reference
**Pinaceae**	mat	pat	review, Mogensen, 1996.
Larix decidua x *leptolepis*	mat	pat*	DeVerno et al, 1991; Scmidt et al. 1987
* 1/6 hybrids mat leakage			
Picea abies	mat	ns	Grivet, et al.,1999
Pinus sp.	mat	pat	Wagner et al. 1987; Neale and Sederoff, 1989
Pinus banksiana x *contorta*	mat*	pat	Wagner et al. 1991
* ~5% pat leakage			
Pinus radiata	ns	pat*	Cato and Richardson, 1996
* ~1% mat leakage			
Pinus taeda	mat	pat	Neal and Sederoff, 1989
Pseudotsuga menziesii	mat*	pat*	Marshall and Neale, 1991; Wagner et al. 1989; Owens and Morris, 1991
* some organelles from pat(mt) or mat(cp) may be included.			
**Taxaceae**			
Taxus baccata	pat	mat	Pennell and Bell, 1988
**Araucariaceae**			
Agathis robusta	pat	pat	Kaur and Bhatnager, 1984
**Cephalotaxaceae**			
Cephalotaxus drupacea	pat	pat	Gianordioli, 1974; Singh, 1961
**Cupressaceae**			
Callitris (4 species)	ns	pat	Sakaguchi, et al.2014
Callitropsis nootkatensis x *Hesperocyparis macrocarpa*	pat	pat	Kou, et al. 2014
Calocedrus decurrens	pat	pat	Neale, Marshall and Harry, 1991
Chamaecyparis obtusa	ns	pat*	Shirashi et al. 2001
* ~2.5% mat leakage			
Chamaecyparis obtusa x pisifera	pat	pat	Kondo, et al., 1998
Chamaecyparis lawsonia	pat	pat	Chesnoy, 1973
Cryptomeria japonica	pat	pat*	Ohba et al. 1971
* some mat leakage			
Cunninghamia konishii	ns	mat	Lu, et al. 2001
Platycladus orientalis	pat	pat	Chesnoy, 1969
Sequoia sempervirens	pat	pat	Neale, Marshall and Sederoff, 1989

mat = maternal; pat = paternal; ns = not studied

Table S3. Sample and sequencing information for Cupressaceae species sequenced in this study

Species	Collection site	Voucher	Library type (bp)	Read len (bp)	Volume (Gb)
Callitropsis nootkatensis	Seattle, WA, USA [b]	Adams 9086 BAYLU	MiSeq 600	2 x 250	4.1 Gb
Cupressus sempervirens	Madrid, Spain [b]	Adams 10388 BAYLU	MiSeq 600	2 x 250	3.9 Gb
Cupressus tonkinensis [a]	Tibet (ex Wang 0026B) [c]	Adams 9300 BAYLU	HiSeq 750	2 x 125	2.6 Gb
Cupressus torulosa	Bhutan (ex Krushforth 1282) [c]	Adams 8136 BAYLU	HiSeq 750	2 x 125	3.2 Gb
Hesperocyparis arizonica	Clifton, AZ [b]	Adams 11672 BAYLU	HiSeq 750	2 x 125	3.6 Gb
Hesperocyparis benthamii	El Chico, Hidalgo, MX [b]	Adams 6879 BAYLU	HiSeq 750	2 x 125	3.6 Gb
Hesperocyparis glabra	Beaver Creek, AZ, USA [b]	Adams 11690 BAYLU	MiSeq 600	2 x 250	3.8 Gb
Hesperocyparis lindleyi	Durango, MX [b]	Adams 14887 BAYLU	HiSeq 750	2 x 125	3.6 Gb
Hesperocyparis lusitanica	Bussaco, Portugal [b]	Adams 7071 BAYLU	HiSeq 750	2 x 125	3.6 Gb
Juniperus communis	var. depressa, NM, USA [b]	Adams 10940 BAYLU	HiSeq 750	2 x 125	2.8 Gb
Xanthocyparis vietnamensis	Vietnam (ex Dan Harder, UK) [c]	Adams 9490 BAYLU	MiSeq 600	2 x 250	3.8 Gb

[a] Originally labeled *Cu. jiangeensis* , but blast analysis of *matK* , *rbcL* , and *trnL-trnF* indicates it is *Cu. tonkinensis*
[b] roadside plant on public land, no permission or license needed
[c] seedling grown from seed by Keith Rushforth, UK, no permission or license needed.

Table S4. GenBank accession numbers for Cupressaceae species sa

Species	Accession number
**Ingroup species**	
Callitropsis nootkatensis	**KP099642**
Cupressus chengiana	KY392754
Cupressus gigantea	KT315754
Cupressus sempervirens	**KP099643**
Cupressus tonkinensis	**MH121046**
Cupressus torulosa	**MH121047**
Hesperocyparis arizonica	**MH121048**
Hesperocyparis benthamii	**MH121049**
***Hesperocyparis glabra* 1**	**KP099644**
Hesperocyparis glabra 2	KX832624
Hesperocyparis lindleyi	**MH121050**
Hesperocyparis lusitanica	**MH121051**
Juniperus cedrus	KT378453
Juniperus communis 1 (*var. communis*)	KR476378
***Juniperus communis* 2 (*var. depressa*)**	**MH121052**
Juniperus formosana	KX832625
Juniperus monosperma	KF866298
Juniperus scopulorum	KF866299
Juniperus virginiana	KF866300
***Xanthocyparis vietnamensis* 1**	**KP099645**
Xanthocyparis vietnamensis 2	KX832629
**Outgroup species**	
Calocedrus formosana 1	AB831010
Calocedrus formosana 2	KX832620
Calocedrus macrolepis	KX832621
Chamaecyparis hodginsii	KX832623
Chamaecyparis lawsoniana	KX832622
Platycladus orientalis	KX832626
Thuja standishii	KX832627
Thujopsis dolabrata	KX832628

Literature cited

Cato, S. A. and T. E. Richardson. 1996. Inter- and intraspecific polymorphism at chloroplast SSR loci and the inheritance of plastids in *Pinus radiata* D. Don. Theor. Appl. Genet. 93: 587-592.

Chesnoy, L. 1969. Sur la participation du gamete male a la constitution du cytoplasme de l'embryon chez le *Biota orientalis* Endl. Rev. Cytol. Biol. Vég. 32: 273-294.

Chesnoy, L. 1973. Sur l'origine paternelle des organites du proembryon du *Chamaecyparis lawsonia* A. Murr (Cupressaceae). Caryologia 25: 223-232.

DeVerno, L. L., P. J. Charest and L. Bonen. 1991 Inheritance of mitochondrial DNA in the conifer *Larix*. Theor. Appl. Genet. 86: 383-388.

Gianordoli, M. 1974. A cytological investigation on gametes and fecundation among *Cephalotaxus drupacea*. In: H. F. Linskens (ed.), Fertilization in higher plants, pp. 221-232. North-Holland, Amsterdam.

Grivet, D., S. Jeandroz and J. M. Favre. 1999 Nad1 b/c intron polymorphism reveals maternal inheritance of mitochondrial genome in *Picea abies*. Theor. Appl. Genet. 99: 346-349.

Kaur, D. and S. P. Bhatnagar. 1984. Fertilization and formation of neocytoplasm in *Agathis robusta*. Phytomorphology 34: 56-60.

Kondo, t., Y. Tsumura, T. Kawahara, and M. Okamura. 1998. Paternal inheritance of chloroplast and mitochondrial DNA in interspecific hybrids of *Chamaecyparis* sp. Breed. Sci. 48: 177-179.

Kou, Y-X., H-Y. Shang, K-S. Mao, Z-H. Li, K. Rushforth and R. P. Adams. 2014. nuclear and cytoplasmic DNA sequence data further illuminate the genetic composition of Leyland Cypresses. J. Am. Soc. Hortic. Sci. 139: 558-566.

Lu, S-Y., C-I., Peng, Y-P. cheng, K-H. Hong and T0Y. Chiang. 2001. Chloroplast DNA phylogeography of *Cunninghamia konishii* (Cupressaceae), an endemic conifer of Taiwan. Genome 44: 797-807.

Marshall, K. A. and D. B. Neale. 1991. The inheritance of mitochondrial DNA in Douglas-fir (*Pseudotsuga menziesii*). Can. J. For. Res. 22: 73-75.

Mogensen, H. L. 1996. The hows and whys of cytoplasmic inheritance in seed plants. Am. J. Bot. 83: 383-404.

Neale, D. B., K. A. Marshall and R. R. Sederoff. 1989. Chloroplast and mitochondrial DNA are paternally inherited in *Sequoia sempervirens* D. Don Endl. Proc. Natl. Acad. Sci. USA 86: 9347-9349.

Neale, D. B., K. A. Marshall and D. E. Harry. 1991. Inheritance of chloroplast and mitochondrial DNA in incense-cedar (*Calocedrus decurrens*). Can. J. For. Res. 21: 717-720.

Neale, D. B. and D. B. Sederoff. 1989. Paternal inheritance of chloroplast DNA and maternal inheritance of mitochondrial DNA in loblolly pine. Theor. Appl. Genet. 77: 212-216.

Ohba, K., M. Iwakawa, Y. Okada and M. Murai. 1971. Paternal transmission of a plastid anomaly in some reciprocal crosses of Sugi, *Cryptomeria japonica* D. Don. Silvae Genetica 20: 101-107.

Owens, J. N. and S. J. Morris. 1991. Cytological basis for cytoplasmic inheritance in *Pseudotsuga menziesii*. II. Fertilization and proembryo development. Am. J. Bot. 78: 1515-1527.

Pennell, R. I. and P. R. Bell. 1988. Insemination of the archegonium and fertilization in *Taxus baccata* L. J. Cell Sci. 89: 551-559.

Sakaguchi, S., Y. tsumura, M. D. Criso, D. M. J. S. Bowman ad Y. Isagi. 2014. Genetic evidence for paternal inheritance of the chloroplast in four Australian *Callitris* species (Cupressaceae). J. For. Res. 19:244-248.

Scmidt, A. E., T. Alden and J-E. Hallgren. 1987. Paternal inheritance of chloroplast DNA in *Larix*. Plant Mol. Biol. 9: 59-64.

Shirashi, S., H. Maeda, T. Toda, K. Seido adn Y. Sasaki. 2001. Incomplete paternal inheritance of chloroplast DNA recognized in *Chamaecyparis obtusa* using an intraspecific polymorphism of the *trnD-trnY* intergenic spacer region. Theor. Appl. Genet. 102: 935-941.

Singh, H. 1961. The life history and systematic position of *Cephalotaxus drupacea* Sieb. Phytomorphology 11: 153-197.

Wagner, D. B., G. R. Furnier, M. A. Saghai-Maroof, S. M. Williams, B. P. dancik and R. W. Allard. 1987. Chloroplast DNA polymorphisms in lodgepole and jack pines and their hybrids. Proc. Natl. Acad. Sci. USA 84: 2097-2100.

Wagner, D. B., D. R. Govindaraju, C. W. Yeatman, and J. A. Pitel. 1989. Paternal chloroplast DNA inheritance in a diallel cross of jack pine (*Pinus banksiana* Lamb.). J. Hered. 80: 483-485.

Wagner, D. B. J. Dong, M. R. Carlson and A. D. Yanchuk. 1991. Paternal leakage of mitochondrial DNA in *Pinus*. Theor. Appl. Genet. 82: 510-514.
